# Evaluating a case-based mental health course for pediatricians in the UAE: a prospective study

**DOI:** 10.3389/fmed.2026.1793546

**Published:** 2026-07-27

**Authors:** Meshal A. Sultan, Ruchita Shah, Hanan A. Al Raeesi, Jwan F. Shekhy

**Affiliations:** 1 Al Amal Psychiatric Hospital, Emirates Health Services, Dubai, United Arab Emirates; 2 College of Medicine, Mohammed Bin Rashid University of Medicine and Health Sciences (MBRU), Dubai, United Arab Emirates; 3 College of Medicine, Gulf Medical University, Ajman, United Arab Emirates; 4 Khorfakkan Hospital, Emirates Health Services, Khorfakkan, United Arab Emirates

**Keywords:** case-based, collaborative care, course, mental health, pediatricians

## Abstract

**Background:**

Pediatricians frequently encounter child and adolescent mental health concerns, yet often report limited training and confidence in managing these presentations. In contexts where access to specialist services is constrained, capacity-building initiatives for generalist clinicians are increasingly important. This pilot study evaluated an 8-week case-based child and adolescent mental health course for pediatricians in the United Arab Emirates using a prospective mixed-methods design.

**Methods:**

All pediatricians at a public secondary-care hospital were invited to participate. Quantitative survey data were collected at baseline, immediately post-course, and at 3–6-month follow-up, assessing attitudes, self-rated knowledge and confidence, and clinical practices related to pediatric mental health. Qualitative data were obtained through post-course and follow-up focus groups. Evaluation was guided by the Kirkpatrick–Barr framework.

**Results:**

Ten pediatricians enrolled; eight completed baseline and follow-up surveys, and nine completed post-course surveys. Satisfaction with the course was high, with all participants rating the content as highly relevant. Group-level analyses demonstrated large improvements in confidence across key competencies (Cohen’s d range: 0.75–2.40), particularly conducting structured mental health screening, discussing mental health concerns with families, and managing mild to moderate mental health difficulties. At follow-up, most participants reported adopting routine screening practices, with 6/8 (75%) reporting routine mental health screening and 5/8 (62.5%) reporting use of standardized tools. Qualitative findings supported increased confidence, practical application of skills, and early integration into clinical workflows, while highlighting ongoing barriers related to time constraints and access to specialist support.

**Conclusion:**

A brief, case-based training program for pediatricians was feasible, highly acceptable, and associated with substantial improvements in self-reported confidence and early practice change sustained at follow-up. These findings support the potential value of structured, practice-oriented training to strengthen frontline pediatric mental health care within collaborative-care models, while underscoring the need for larger controlled studies incorporating objective clinical outcomes.

## 1 Introduction

Child and adolescent mental health is a growing global health priority, with a substantial proportion of young people experiencing psychiatric disorders that remain unrecognized or untreated (1); World Health Organization (WHO), (2, 3). Estimates suggest that more than half of pediatric primary care visits involve psychological or behavioral concerns, and pediatricians are often both the first point of contact and the primary prescribers of psychotropic medications for children and adolescents (1, 4). At the same time, specialist child and adolescent psychiatry and psychology services remain limited worldwide, including in many high-income countries, contributing to marked treatment gaps and delayed access to evidence-based care (1, 5, 6); World Health Organization (WHO), (3).

To address these gaps, there has been increasing emphasis on models that build the capacity of generalist providers to identify and manage common mental health problems in youth (1); World Health Organization (WHO), (2, 3). Within a stepped-care framework, pediatricians are well-placed to provide first-line assessment, psychoeducation, and brief interventions for mild to moderate conditions, while referring more complex cases to specialist services (1, 6). Interdisciplinary collaborative-care models—linking pediatricians with child psychiatrists, psychologists, and other mental health professionals—have been associated with improved accessibility, quality, and cost-effectiveness of care (4, 6); World Health Organization (WHO), (3). By formalizing co-management structures, shared care pathways, and regular case discussions, such models aim to bridge the gap between rising pediatric mental health needs and the limited availability of specialized services.

Despite this central role, pediatricians and other primary care physicians often receive limited training in child and adolescent mental health during undergraduate and postgraduate education (4, 7, 8). More recent work has highlighted persistent gaps in behavioral and mental health competencies among pediatric trainees, including limited preparedness to assess and manage common emotional and behavioral conditions in children and adolescents (9). Additional evidence suggests that acquisition of these skills during training is variable, with important differences in where and how pediatric trainees gain experience in behavioral and mental health care (10). Together, these findings reinforce the need for structured and practice-oriented educational initiatives that strengthen frontline clinicians’ competence and confidence in pediatric mental health care. International literature similarly highlights gaps in knowledge, low confidence in managing behavioral and emotional disorders, and discomfort with prescribing psychotropic medications for children (7, 8, 11).

In some local healthcare settings, these challenges are compounded by institutional policies that restrict pediatricians’ privileges to initiate or manage psychotropic medications in children, despite their role as first-line providers. Continuing education bodies such as the Accreditation Council for Continuing Medical Education (ACCME) emphasize that effective continuing professional development should be needs-based, interactive, and designed using evidence-informed educational strategies that support behavior change in practice Accreditation Council for Continuing Medical Education (ACCME), (12). However, many existing initiatives are short, didactic, and insufficiently aligned with clinicians’ real-world challenges (7).

In Canada, surveys of rural and remote family physicians demonstrated strong interest in practical child mental health training, particularly around attention-deficit/hyperactivity disorder (ADHD), anxiety, and disruptive behavior disorders (8, 11). A systematic review by Gotovac et al. (7) found that a range of training programs exist for primary care providers, but few have been rigorously evaluated across multiple outcome levels. The Practitioner Training in Child and Adolescent Psychiatry (PTCAP), an 8-h Continuing Medical Education (CME)-accredited course, is one of the better-studied interventions. In a cluster-randomized trial, Espinet et al. (13) reported that PTCAP significantly improved physicians’ confidence in managing common mental health conditions in youth, although gains in factual knowledge and attitudes were more modest. These findings echo broader evidence from the continuing education literature that short-term training can increase self-efficacy, but more intensive or sustained interventions, coupled with ongoing support and mentorship, may be needed to consolidate skills and influence long-term practice Accreditation Council for Continuing Medical Education (ACCME), (7, 12).

In the Middle East, early work by El Rufaie (14, 15) drew attention to the extent of “hidden psychiatric morbidity” in primary care—particularly depression and anxiety—that often goes undetected by general practitioners, leading to diagnostic delays and unnecessary investigations. He underscored the need for context-sensitive training programs tailored to primary care providers’ workloads, cultural context, and system constraints (14, 15). These observations remain highly relevant in the United Arab Emirates (UAE), where pediatricians and family physicians frequently serve as the only point of contact for families concerned about their children’s emotional and behavioral difficulties (4).

This need is further underscored by evidence that child and adolescent mental health problems are common in the UAE. In a population-based study of adolescents in the UAE, anxiety disorders were identified in 28% of participants (16), while a community survey in Al Ain reported an overall child psychiatric morbidity rate of 22.2% (17). In addition, a recent needs assessment in Dubai demonstrated that only about one-third of surveyed primary care physicians had formal training in child and adolescent psychiatry, and more than half reported feeling unprepared to manage child mental health presentations (4). Respondents described barriers such as limited knowledge, stigma, time constraints, and difficulties accessing specialist consultation. The study recommended integrated care pathways and targeted, practice-oriented training as key strategies to improve early detection and frontline management of pediatric mental health problems in the UAE (4). These findings helped inform the selection of course topics, which focused on common and practical presentations relevant to frontline pediatric care, including ADHD, anxiety, autism spectrum disorder, behavioral challenges, sleep difficulties, emotional dysregulation, and referral/collaboration pathways.

In 2025, Emirates Health Services (EHS) launched 15 specialized mental health clinics embedded within primary care centers across six emirates, staffed by trained family physicians providing early diagnostic and therapeutic services (18). EHS leadership has emphasized the importance of equipping generalist clinicians—including pediatricians—with skills in early identification and first-line management as part of a broader integrated mental health strategy (18); World Health Organization (WHO), (3).

Within this context, there is a critical need for structured, evaluated training programs that strengthen pediatricians’ competencies in child and adolescent mental health and support collaborative care with specialist services. The current study responds to this need by developing and evaluating an 8-week case-based mental health course for pediatricians at Khorfakkan Hospital, an Emirates Health Services facility in the UAE. The course, facilitated by child and adolescent psychiatrists from Al Amal Psychiatric Hospital, is designed to be practice-oriented and locally relevant, with weekly sessions covering topics such as anxiety, ADHD, autism spectrum disorder, behavioral difficulties, sleep problems, and emotional dysregulation, as well as strategies for interdisciplinary collaboration and referral.

To evaluate the impact of the course, we employ the Kirkpatrick–Barr framework, which assesses training effectiveness across four levels: learners’ reactions (satisfaction), learning (knowledge and skills), behavior (changes in clinical practice), and results (impact on patient care and service delivery) (19). This framework, widely used in health professions education, allows for a comprehensive assessment of both short-term learning outcomes and intermediate practice changes.

The aim of this prospective study is to examine the effectiveness of the 8-week case-based child and adolescent mental health course in improving pediatricians’ knowledge, confidence, and self-reported practice related to pediatric mental health care, and to explore preliminary indications of impact on patient- and system-level outcomes within a collaborative-care context in the UAE.

## 2 Materials and methods

### 2.1 Study design and framework

This pilot study used a prospective mixed-methods design to evaluate an 8-week case-based child and adolescent mental health course for pediatricians. Quantitative survey data were collected at baseline, immediately after the course, and at 3–6-month follow-up, while qualitative data were collected through post-course and follow-up focus groups. A mixed-methods approach was chosen to allow quantitative assessment of changes in self-reported knowledge, confidence, and practice, while also capturing participants’ experiences, perceived barriers and facilitators, and contextual examples of how learning was applied in clinical settings.

The evaluation was guided by the Kirkpatrick–Barr framework (19), examining:

- Level 1 – Reaction: participants’ satisfaction and perceived relevance;
- Level 2 – Learning: changes in knowledge and confidence;
- Level 3 – Behavior: self-reported changes in clinical practice;
- Level 4 – Results: perceived impact on patient care and service delivery.

Given the reliance on self-reported survey and focus group data, Level 3 and Level 4 outcomes in this study were interpreted as self-reported practice change and perceived patient or service-level impacts, rather than objective clinical outcomes. The Kirkpatrick–Barr framework was therefore used to guide evaluation of participants’ reactions, learning, and reported early behavioral and service-related changes, while recognizing that higher-level outcomes were assessed indirectly.

### 2.2 Setting

The study was conducted at Khorfakkan Hospital, an Emirates Health Services (EHS) facility in the United Arab Emirates, where pediatricians provide frontline inpatient and outpatient care to children and adolescents. The course was designed and facilitated by child and adolescent psychiatrists from Al Amal Psychiatric Hospital (EHS).

### 2.3 Participants

The study population comprised pediatricians practicing at Khorfakkan Hospital during the study period.

Inclusion criteria were:

- Doctor of Medicine (MD) or equivalent with specialization in pediatrics;
- Currently practicing at Khorfakkan Hospital;
- Ability to attend the majority of the eight course sessions;
- Willingness to complete the evaluation surveys.

Exclusion criteria were inability to commit to at least 6 of 8 sessions or refusal to participate in the evaluation component. Clinicians who attended the course but did not complete the research measures were excluded from analyses but could still participate in the educational sessions.

All eligible clinicians (10 in total) were invited through departmental meetings and email. Ten pediatricians enrolled in the course. Of these, eight (80%) completed the pre-course (baseline) survey, nine (90%) completed the immediate post-course survey, five took part in the end-of-course focus group, six participated in the follow-up focus group, and eight (80%) completed the follow-up survey.

### 2.4 Educational intervention

The intervention was an 8-week case-based child and adolescent mental health course tailored to the needs of pediatricians in Khorfakkan. Sessions lasted 60 min and were delivered either in person or online via Microsoft Teams. Each session combined:

- a brief evidence-informed overview of the topic;
- one or more locally relevant clinical vignettes;
- guided case discussion;
- practical strategies for assessment, management, and collaboration with mental health services.

The schedule was:

- Recognizing and Managing ADHD in Primary Care – 2 June 2025 (in person) – Facilitator: M.A.S.
- Anxiety Disorders in Children and Adolescents – 12 June 2025 (online, MS Teams) – Facilitator: M.A.S.
- Autism Spectrum Disorder: Identification and Early Support – 19 June 2025 (online, MS Teams) – Facilitator: R.S.
- Behavioral Challenges and Disruptive Behaviors – 26 June 2025 (online, MS Teams) – Facilitator: R.S.
- Emotional Dysregulation and Irritability – 7 July 2025 (in person) – Facilitator: M.A.S.
- School Refusal, Academic Struggles, and Processing Issues – 17 July 2025 (online, MS Teams) – Facilitator: R.S.
- Sleep and Mental Health – 24 July 2025 (online, MS Teams) – Facilitator: R.S.
- When to Refer Promptly and How to Collaborate – 31 July 2025 (in person) – Facilitator: M.A.S.

The selection of topics was based on discussions between the course facilitators and pediatricians in Khorfakkan Hospital to identify relevant and important areas in child and adolescent mental health. Furthermore, content drew on evidence-based guidance for integrating child mental health into primary care and on local needs identified in a recent Dubai-based needs assessment (1, 4); World Health Organization (WHO), (2, 3).

### 2.5 Data collection and time points

The study sample comprised pediatricians participating in the 8-week case-based child and adolescent mental health course delivered at Khorfakkan Hospital. Quantitative data were collected through pre-course, post-course, and follow up surveys. Qualitative data were collected through two semi-structured, in-person focus groups conducted at Khorfakkan Hospital: one at the end of the course and one at follow-up. These discussions explored participants’ experiences of the course, perceived strengths and limitations, application of learning to clinical practice, barriers and facilitators to implementation, and suggestions for improvement. The follow-up focus group was audio-recorded with participants’ permission, transcribed, and anonymized prior to analysis.

Data collection points were as per the following:

- Baseline (pre-course) survey – 2 June 2025

Administered immediately before the first session, this questionnaire assessed:

- demographics and professional profile (age group, gender, years in practice, role);
- prior mental health training and exposure to psychiatry;
- baseline self-rated knowledge and confidence in managing common pediatric mental health conditions;
- attitudes toward pediatric mental health care;
- current screening and referral practices;
- perceived barriers and training needs.
- Immediate post-course survey – 31 July 2025

Completed at the end of the eighth session, this survey captured:

- Level 1 (Reaction): satisfaction with course content, format, facilitation, and perceived relevance;
- Level 2 (Learning): self-rated changes in knowledge and confidence using the same items as baseline;
- Intended behavior change: specific ways participants planned to modify their practice (e.g., using screening tools, applying brief counseling strategies, increasing collaboration with psychiatry).
- Post-course focus group – 4 August 2025

A semi-structured in-person focus group explored participants’ experiences of the course, perceived strengths and limitations, early examples of applying new knowledge or skills, and suggestions for improving future iterations.

- Follow-up survey and focus group – 3–12 November 2025

A follow-up survey (open from 3 to 12 November 2025) and a focus group on 3 November 2025 assessed:

- Level 3 (Behavior): implementation of intended practice changes (e.g., screening routines, brief interventions, number and management of mental health cases, collaboration with psychiatry);
- Level 4 (Results): perceived impact on patient care (e.g., symptom improvement, parental satisfaction) and on service delivery (e.g., appropriateness and timeliness of referrals, any new protocols);
- ongoing barriers and facilitators to practice change;
- additional training and support needs.

## 3 Measures

### 3.1 Quantitative measures

- Demographics and professional characteristics: age group, gender, years since qualification, years in pediatrics, role, and estimated proportion of caseload involving mental or behavioral concerns.
- Prior training: history of courses, workshops, or rotations in psychiatry or child and adolescent mental health (yes/no and brief description).
- Knowledge and familiarity: Likert-scale items rating familiarity with key conditions, treatment principles, and selected guidelines/resources (e.g., WHO guidance). A composite knowledge/familiarity score was calculated by averaging relevant items.
- Confidence: for common clinical scenarios (e.g., depression, anxiety, ADHD, disruptive behavior, sleep problems, autism spectrum disorder), participants rated their confidence in assessment, counseling, and management on 5-point Likert scales (1 = not at all confident to 5 = very confident). Domain-specific and overall confidence scores were computed.
- Attitudes: agreement with statements about the role of pediatricians in mental health (e.g., “It is appropriate for pediatricians to manage mild mental health problems in primary care”) on five-point scales.
- Practice patterns and collaboration: items assessing routine screening practices, use of brief psychosocial interventions, number and type of mental health referrals in the preceding 6 months, and frequency of consultation with mental health specialists. Follow-up surveys included checklists of specific practice changes adopted since the course.
- Satisfaction and reaction (post-course only): overall satisfaction, perceived relevance, quality of case discussions, and facilitator effectiveness, rated on Likert scales, plus open-ended comments.

### 3.2 Qualitative data

Open-ended questions within the surveys and the semi-structured focus groups elicited more detailed descriptions of:

- experiences of the course;
- examples of changed practice;
- patient or family responses;
- barriers and facilitators to implementation;
- suggestions for further training or service changes.

### 3.3 Variables and outcomes

Key independent variables included demographic characteristics, years in practice, prior mental health training (yes/no), baseline knowledge and confidence scores, and course attendance (number of sessions attended).

Primary outcomes were:

- Level 2 (Learning): change in knowledge/familiarity and confidence scores from baseline to immediate post-course and from baseline to follow-up;
- Level 3 (Behavior): adoption of new screening, counseling, and management practices and changes in self-reported referral patterns and collaboration at follow-up.

Secondary outcomes included:

- Level 1 (Reaction): satisfaction and perceived usefulness;
- Level 4 (Results): participants’ perceptions of improvements in patient care and system processes, and any observable changes in service indicators where available.

### 3.4 Data analysis

Quantitative analyses were conducted using standard statistical software (SPSS).

• Descriptive statistics summarized participant characteristics, satisfaction ratings, and practice patterns (means and standard deviations or medians and interquartile ranges for continuous variables; counts and percentages for categorical variables).
• Pre–post comparisons examined changes in key outcomes:
  • Changes in self-reported knowledge and confidence in different child and adolescent mental health (CAMH) areas, practice behaviors and follow-up outcomes were summarized descriptively.
  • Pre–post changes in confidence in specific skills were assessed using paired *t*-tests for approximately normally distributed variables where data could be reliably matched. Group level analysis was conducted using independent samples *t*-test; effect sizes (Cohen’s d, corrected with Hedges’ g) and 95% confidence intervals quantified magnitude of change.
  • Given small sample sizes and ordinal outcome scales, non-parametric Mann–Whitney U tests were conducted as sensitivity analyses to examine the robustness of the findings.
  • Changes in proportions (e.g., percentage routinely screening for mental health concerns) were described and, where cell sizes allowed, tested with McNemar’s test.
• Exploratory analyses assessed whether improvements varied by baseline characteristics (e.g., prior mental health training, years in practice), using simple correlations or two-group comparisons (independent *t*-tests), recognizing the limited power of a small sample.
• Qualitative data were analyzed using Braun and Clarke’s six-step thematic analysis (20). Researchers first familiarized themselves with the data through review of notes, recordings, and written responses from both the post-course and follow-up sessions. Initial codes were generated from meaningful segments of text and grouped into broader themes reflecting patterns across participants’ experiences. Themes were reviewed and refined through discussion between two researchers and aligned with the Kirkpatrick–Barr evaluation levels (Reaction, Learning, Behavior, and Results) to examine changes from post-course intentions to follow-up implementation and outcomes.

The qualitative component was exploratory and based on the available participants from this single-site course cohort. Five pediatricians participated in the end-of-course focus group and six participated in the follow-up focus group. Given the small and bounded sample, the qualitative data were intended to provide depth, triangulation, and contextual interpretation of the quantitative findings rather than to establish data saturation. Qualitative data were analyzed using an inductive thematic approach, with independent coding by two researchers followed by discussion and refinement of themes.

Given the single-site setting and small cohort, emphasis was placed on effect sizes, confidence intervals, and triangulation of quantitative and qualitative data, rather than solely on *p*-values.

### 3.5 Ethical considerations

The study protocol and data collection instruments were reviewed and approved by the Ministry of Health and Prevention (MOHAP) Research Ethics Committee, United Arab Emirates (Approval Reference MOHAP/DXB-REC/J-J.J/No. 124/2025). The approval covered both Al Amal Psychiatric Hospital and Khorfakkan Hospital as study sites.

All participants received written information about the study and provided written informed consent before enrollment. Participation in the evaluation was voluntary and independent of employment or mandatory education requirements. Surveys used anonymous study ID codes. Focus group transcripts and any illustrative quotations were de-identified prior to analysis. Data were stored on password-protected computers accessible only to the research team. Participants were informed that they could withdraw at any time without penalty and that aggregate results might be disseminated through scientific publications and presentations.

## 4 Results

### 4.1 Quantitative analysis

#### 4.1.1 Pre-course

At baseline, eight general pediatricians completed the pre-course survey. The mean age was 41.0 years (SD = 5.74), with most respondents identifying as female (5/8, 62.5%). Participants reported substantial experience in practice, with a mean of 10.9 years (SD = 4.67) in their profession and 4.3 years (SD = 2.71) in their current role. The majority reported Arabic as their primary language (7/8, 87.5%). Only two participants (25%) had ever attended a workshop, course, or training specifically focused on child or adolescent mental health, although most reported some psychiatry exposure during medical school or residency: half had both formal teaching and clinical experience (4/8, 50%), a quarter had teaching only (2/8, 25%), and a quarter reported no formal exposure (2/8, 25%). Mental and behavioral health concerns already formed a non-trivial portion of their clinical work: five respondents (62.5%) indicated that less than 20% of their weekly practice involved such presentations, while one (12.5%) reported 26%–50% and two were unsure or did not respond (Table 1). Self-rated overall competence in managing child and adolescent mental health was generally modest, with most participants describing their competence as moderate (5/8, 62.5%), two as low (25%), and one as very low (12.5%). Similarly, when asked to rate their current knowledge, half rated it as good (4/8, 50%), three as fair (37.5%), and one as poor (12.5%).

**TABLE 1:** Baseline participant characteristics (*n* = 8).

Characteristic	Mean (SD)	*n* (%)
Age	41.0 (5.74)	–
Female	5 (62.5)	–
Years in practice	10.9 (4.67)	–
Years in current role	4.3 (2.71)	–
Primary language Arabic	–	7 (87.5)
Prior CAMH-specific training	–	2 (25.0)
Any psychiatry exposure during training	–	6 (75.0)
Mental/behavioral health < 20% of weekly practice	–	5 (62.5)
Cadre/role: general pediatrician	–	8 (100)

SD, standard deviation; CAMH, Child and Adolescent Mental Health.

Baseline confidence ratings across specific scenarios suggested important skill gaps. On the (Likert-type) confidence scale, respondents reported the lowest confidence for conducting structured mental health screening using tools such as the Strengths and Difficulties Questionnaire (mean = 1.98, SD = 0.90) and for managing mild to moderate mental health problems (mean = 2.29, SD = 1.11). In comparison, participants felt relatively more confident explaining diagnoses or treatment plans (mean = 2.86, SD = 1.46) and, in particular, in referring to mental health specialists (mean = 3.57, SD = 1.27) and coordinating care with them (mean = 3.29, SD = 1.11). Overall, these data indicated a pattern of greater comfort with referral and coordination roles than with frontline screening, assessment, and active management.

Attitudinal responses demonstrated broad recognition of the importance of child and adolescent mental health in pediatric practice, with most respondents agreeing that pediatricians can positively impact patients’ mental health and that addressing mental health concerns forms part of their professional role, although views on managing mild mental health conditions within primary care were mixed (Table 2). Routine practice reflected limited formalization, with fewer than half of participants reporting regular screening for mental health concerns and none using standardized screening tools; screening, when conducted, relied largely on informal clinical approaches. Most participants had made recent referrals to mental health or allied professionals. The most frequently reported barriers were lack of training, limited access to specialists, and stigma, while free-text responses highlighted a need for structured guidance, standardized assessment tools, and practical support to strengthen frontline management.

**TABLE 2:** Baseline attitudes, practices, and perceived barriers.

Domain	*n* (%)
Pediatricians can positively impact mental health (attitude)	6/7 (85.7)
Addressing mental health is part of the role (attitude)	4/7 (57.1)
Pediatricians should manage mild conditions (attitude)	3/7 (42.9)
Routinely screen for mental health concerns (practice)	3/8 (37.5)
Use standardized screening tools (practice)	0/8 (0)
Lack of training (barrier)	3/7 (42.9)
Limited specialist access (barrier)	3/7 (42.9)
Stigma (barrier)	3/7 (42.9)

#### 4.1.2 Post-course

##### 4.1.2.1 Post-course satisfaction and perceived relevance

Nine participants completed the immediate post-course survey. Overall satisfaction with the training was very high: seven respondents (77.8%) reported being *very satisfied* and two (22.2%) *satisfied*. All participants rated the content as either *extremely relevant* (3/9, 33.3%) or *very relevant* (6/9, 66.7%) to their clinical practice. The format and delivery of the training were also rated very positively, with eight participants (88.9%) describing them as *excellent* and one (11.1%) as *good*, indicating a strong perceived fit between course design and learner needs.

##### 4.1.2.2 Post-course self-rated knowledge and confidence in CAMH areas

Post-course, all participants rated their knowledge in child and adolescent mental health as good or excellent. Self-rated improvement in knowledge and confidence were assessed using a five-point Likert-type scale (1 indicating “No improvement” to 5 indicating “Very much improved”). Perceived improvements in knowledge were high across all targeted conditions, with the largest improvement reported for suicide risk assessment and consistently strong gains across neurodevelopmental, emotional, behavioral, and school-related presentations (Table 3). When examined categorically, the majority of participants reported high levels of improvement (significant to very much improved) across all domains, with minimal reports of no or slight change. Self- perceived improvement in confidence mirrored these findings, with uniformly high improvements reported across all clinical domains.

**TABLE 3:** Post-course self-reported improvements in knowledge and confidence (*n* = 9).

Domain	Self-reported improvement in knowledge			Self-reported improvement in knowledge* mean (SD)	Self-reported improvement in confidence			Self-reported improvement in confidence* mean (SD)
	No/minimal change *n* (%)	Moderate improvement *n* (%)	High improvement *n* (%)		No/minimal change *n* (%)	Moderate Improvement *n* (%)	High improvement *n* (%)	
ADHD	0 (0.00%)	1 (11.11%)	8 (88.89%)	4.11 (0.60)	0 (0.00%)	1 (11.11%)	8 (88.89%)	4.11 (0.60)
Anxiety disorders	0 (0.00%)	1 (11.11%)	8 (88.89%)	4.11 (0.60)	0 (0.00%)	1 (11.11%)	8 (88.89%)	4.33 (0.70)
Autism spectrum disorder	1 (11.11%)	2 (22.22%)	6 (66.67%)	3.87 (0.99)	1 (11.11%)	0 (0.00%)	8 (88.89%)	4.00 (0.87)
School refusal/academic struggles	0 (0.00%)	1 (11.11%)	8 (88.89%)	4.22 (0.67)	1 (11.11%)	0 (0.00%)	8 (88.89%)	4.11 (1.27)
Emotional dysregulation/irritability	1 (11.11%)	1 (11.11%)	7 (77.78%)	3.78 (1.20)	0 (0.00%)	2 (22.22%)	7 (77.78%)	4.22 (0.83)
Behavioral challenges	0 (0.00%)	2 (22.22%)	7 (77.78%)	4.00 (0.71)	0 (0.00%)	1 (11.11%)	8 (88.89%)	4.22 (0.67)
Sleep difficulties	1 (11.11%)	1 (11.1%)	7 (77.78%)	3.89 (0.93)	1 (11.11%)	0 (0.00%)	8 (88.89%)	4.00 (0.87)
Suicide risk assessment	0 (0.00%)	0 (0.00%)	9 (100.00 %)	4.58 (0.53)	0 (0.00%)	0 (0.00%)	9 (100.00 %)	4.56 (0.53)

ADHD, attention-deficit/hyperactivity disorder. *As assessed during post-course survey; Scores range from 1 (no improvement) to 5 (very much improved).

Self-reported improvements in knowledge or confidence were not significantly associated with age, years of experience, gender, or prior training.

##### 4.1.2.3 Post-course change in confidence in specific skills

Beyond these global ratings, comparisons of pre- and post-course confidence scores showed statistically significant improvements in several specific clinical skills.

Group-level analyses (Table 4a) demonstrated large to very large improvements in self-reported confidence across most competency domains following the intervention (Cohen’s d range: 0.75–2.40). Small-sample–corrected effect sizes (Hedges’ g) indicated large to very large group-level improvements across most competency domains (g range: 0.71–2.27). The largest effects were observed for conducting structured mental health screenings and managing mild to moderate mental health difficulties. Confidence intervals were wide, reflecting small samples. Although for one domain (referral to specialists) did not reach statistical significance, effect sizes suggest potentially meaningful changes that warrant further investigation, with uncertainty reflected in wide confidence intervals.

**TABLE 4a:** Pre–post changes in confidence for specific clinical skills (group-level analysis).

Domain	Pre mean (SD), n	Post mean (SD), *n*	Mean difference (post–pre)	*t* (df)	*P*	Cohen’s d	Hedges’ g (95% CI)
Conducting mental health screenings using a validated tool	1.86 (0.90), *n* = 7	3.89 (0.78), *n* = 9	2.03	6.04 (14)	<0.001*	2.40	2.27 (0.99, 3.55)
Discussing mental health concerns with families	2.71 (1.11), n = 7	4.44 (0.53), n = 9	1.73	4.60 (14)	0.001*	1.92	1.82 (0.67, 2.97)
Managing mild/moderate mental health issues	2.29 (1.11), *n* = 7	4.22 (0.67), *n* = 9	1.93	4.92 (14)	<0.001*	1.99	1.89 (0.72, 3.06)
Explaining diagnoses or treatment plans	2.86 (1.46), *n* = 7	4.44 (0.53), *n* = 9	1.58	3.22 (14)	0.006*	1.39	1.32 (0.29, 2.35)
Employing CBT techniques^#^	–	4.11 (0.60), n = 9	–	–	–	–	–
Providing advice to parents (behavioral management)	2.71 (1.50), *n* = 7	4.33 (0.71), *n* = 9	1.62	3.01 (14)	0.010*	1.32	1.25 (0.25, 2.25)
Referring to mental health specialists	3.57 (1.27), *n* = 7	4.38 (0.74), *n* = 8	0.81	1.75 (13)	0.104	0.75	0.71 (−0.32, 1.74)
Coordinating care with mental health professionals	3.29 (1.11), *n* = 7	4.33 (0.50), *n* = 9	1.04	2.90 (14)	0.012*	1.19	1.13 (0.15, 2.11)

For group-level analyses, valid responses were reported separately for each domain (“*n*” varies per variable), reflecting the number of participants with complete data for that specific skill at each time point; SD, standard deviation; CBT, Cognitive Behavioral Therapy. Effect sizes reported as Hedges’ g. ^#^CBT techniques were not assessed at baseline; therefore, no pre–post comparison is available for this domain. *P < 0.05 (two-tailed).

Sensitivity analyses using Mann–Whitney U tests yielded the same pattern of results, with post-training scores consistently higher than pre-training scores across most domains. While statistical significance was attenuated for some domains (“referral to specialists” and Coordinating care with mental health professionals) in the non-parametric analyses, the direction and magnitude of effects remained consistent.

Paired-samples *t*-tests were conducted for the subset of participants (*n* = 4) for whom pre- and post-intervention data could be reliably matched (Table 4b). Due to missing identifying codes on follow up, paired comparisons were not possible for all participants. Statistically significant improvements were observed in conducting mental health screenings [Mean difference = 2.25, *t*(3) = −4.70, *p* = 0.018] and discussing mental health concerns with families [Mean difference = 1.50, *t*(3) = −5.20, *p* = 0.014].

**TABLE 4b:** Pre–post changes in confidence for specific clinical skills (paired-samples analysis) (*n* = 4).

Domain	Pre mean (SD)	Post mean (SD)	Mean difference (post–pre)	*t* (df)	*P*
Conducting mental health screenings using a validated tool	1.75 (0.96)	4.00 (0.00)	2.25	−4.70 (3)	0.018*
Discussing mental health concerns with families	3.00 (1.16)	4.50 (0.58)	1.50	−5.20 (3)	0.014*
Managing mild/moderate mental health issues	2.50 (1.29)	3.75 (0.50)	1.25	−1.46 (3)	0.239
Explaining diagnoses or treatment plans	3.25 (1.71)	4.25 (0.50)	1.00	−1.23 (3)	0.308
Providing advice to parents (behavioral management)	3.25 (1.71)	4.00 (0.82)	0.75	−0.73 (3)	0.519
Referring to mental health specialists	3.75 (1.50)	4.00 (0.82)	0.25	−0.29 (3)	0.789
Coordinating care with mental health professionals	3.50 (1.29)	4.25 (0.50)	0.75	−1.57 (3)	0.215

SD, standard deviation. Scores range from 0 (no confidence) to 5 (very confident). *P < 0.05 (two-tailed).

Taken together, these findings indicate both broad perceived improvement and statistically robust gains in key competencies targeted by the course.

##### 4.1.2.4 Intended practice changes

Behavioral intentions following the course were uniformly positive. All participants (100%) stated that they intended to make changes in their clinical practice as a result of the training. The most frequently endorsed changes were to conduct more regular mental health screenings (seven responses), initiate more routine conversations about mental health with patients and families (seven responses), and make referrals more confidently and appropriately (seven responses).

Many respondents reported plans to begin using standardized screening tools such as the Vanderbilt ADHD Rating Scales (VARS), Modified Checklist for Autism in Toddlers (M-CHAT), and Strengths and Difficulties Questionnaire (SDQ) (six responses), enhance collaboration with mental health specialists (five responses), and strengthen liaison with schools (three responses) to support children more effectively. In free-text elaborations, five participants described concrete strategies for applying course content, including combining screening with brief communication-based interventions, and using structured tools to guide decisions about intervention and referral.

##### 4.1.2.5 Qualitative findings

Participants described the course as practical and relevant to pediatric care. One participant noted that it “covered common presentations in the pediatric setting” (P1, post-course focus group), while another reported becoming “more alert for early identification” (P2, post-course focus group). At follow-up, participants also described practice change, including “use of screening for autism, which helped in timely referral” (P3, follow-up focus group). Barriers to implementation were also noted, particularly “lack of time” (P4, follow-up focus group) and challenges related to access to specialized services.

Participants’ comments further illustrated the perceived usefulness and areas for refinement. They highlighted a wide range of valuable course components, including guidance on when to refer to psychiatric services, ADHD and ASD content, early signs of child abuse, school refusal, anxiety and behavioral disturbances, diagnostic and severity tools, templates and referral processes, and decision-making around school-related cases.

Suggested improvements centered on expanding in-person contact for better interaction, clarifying inter-hospital linkage processes, providing more detailed autism content, and adding a dedicated practical session focused solely on case discussions. Respondents described the training as very helpful for upskilling, informative and comprehensive, and expressed interest in ongoing access to recorded courses and shared folders containing the tools introduced. Several emphasized the importance of sustaining and institutionalizing such training to support long-term changes in practice. All participants indicated that they would recommend this training to a colleague, reinforcing its acceptability and perceived value.

#### 4.1.3 Three-month follow-up: practice, patient, and system-level changes

All eight clinicians who completed the 3-month follow-up reported changes in how they screen for and manage pediatric mental health concerns. Most implemented routine screening, discussed emotional/behavioral concerns with families, and began using standardized tools (VARS, M-CHAT, SDQ). Clinicians also adapted referral pathways, integrated screening into workflows, and initiated case discussions (Table 5).

**TABLE 5:** Three-month follow-up: practice, patient, and system-level changes (*n* = 8).

Domain	Key changes/outcomes	*n* (%)
Practice changes	Routine mental health screening	6 (75%)
	Discuss emotional/behavioral concerns with families	6 (75%)
	Use standardized screening tools (VARS, M-CHAT, SDQ)	5 (62.5%)
	Adapt referral pathways	5 (62.5%)
	Integrate screening into clinic workflow	4 (50%)
	Initiate case discussions with colleagues/specialists	4 (50%)
	Develop patient/family education materials	2 (25%)
Patient-level outcomes	Increased clinician confidence and proactive care	7 (87.5%)
	Observed improvements in patient mental health symptoms	7 (87.5%)
	Improvements in school attendance/performance	6 (75%)
	Greater family satisfaction and engagement	6 (75%)
	More timely and appropriate referrals	7 (87.5%)
System-level changes	Introduced new protocols or tools (e.g., VARS, M-CHAT)	5 (62.5%)
	More structured collaboration with mental health specialists	5 (62.5%)
	New or modified integrated care pathways	3 (37.5%)
	Ongoing efforts toward coordinated early identification/management	5 (62.5%)
Ongoing barriers	Limited time in clinic	8 (100%)
	Limited access to specialists	3 (37.5%)
Support Needs	Refresher training, case-based discussions	4 (50%)

VARS, Vanderbilt ADHD Rating Scales; M-CHAT, Modified Checklist for Autism in Toddlers; SDQ, Strengths and Difficulties Questionnaire.

Paired pre–post data were available for four respondents only. At baseline, none reported using structured screening tools (such as SDQ and VARS), whereas all four reported screening using structured tools at follow-up. McNemar’s exact test indicated a consistent change in screening behavior across participants (*p* = 0.063, two-sided). Although statistical significance was not reached due to the very small paired sample, the effect size was maximal, with a 100% absolute increase in screening uptake (risk difference = 1.00) and all respondents changing in the same direction following training.

Patient-level outcomes were positive, with most clinicians reporting increased confidence, improved care, better patient mental health, greater family satisfaction, and more timely referrals. System-level changes included introducing new protocols and tools, more structured collaboration with mental health specialists, and development or refinement of integrated care pathways (Table 5).

Ongoing barriers included limited time, restricted specialist access, and the need for refresher training or case-based discussions. Suggested supports included protected time, easier access to psychiatry, periodic review of materials, and regular case-review meetings (Table 5).

### 4.2 Qualitative analysis

As part of the course evaluation, two semi-structured focus group discussions were conducted. A post-course focus group was held on 4 August 2025, with five pediatricians who participated in the course. To assess sustained changes in practice and perceived impact, a follow-up focus group was conducted 3 months later, on 3 November 2025, with six pediatricians (with some overlap from the original group). The follow-up deliberately built on the end-of-course discussion and emphasized real-world implementation, enablers and barriers within clinic workflows, and ongoing support needs (e.g., mentorship and joint clinics).

Both discussions were guided by a protocol aligned with the Kirkpatrick–Barr evaluation framework (19, 21), covering participants’ experiences, perceived learning, behavioral intentions/changes, barriers to implementation, and suggestions for improvement. Each session was facilitated by the course director (M.A.S.) and documented via handwritten notes and an audio recording. Following each session, both course facilitators (M.A.S. and R.S.) jointly reviewed materials, discussed analytic decisions, and resolved discrepancies as an investigator-triangulation step to enhance trustworthiness. The follow-up protocol additionally prompted reflections on sustained change (e.g., cases influenced by the course, adjustments to clinic processes, and perceived patient/family outcomes).

Qualitative analysis followed Braun and Clarke’s six-step thematic analysis (20). For the post-course dataset, the steps were applied as planned. For the follow-up dataset, the same steps were repeated and a cross-time comparison examined convergence and change from intention (immediate post-course) to observed behavior and perceived results (3-month follow-up): (1) familiarization, with review of notes, recordings, and reflections within 3 days of each session to preserve context; (2) generation of initial codes, grouping responses against the guide and, at follow-up, tagging whether they evidenced sustained behavior change or outcomes; (3) theme development aligned to Kirkpatrick–Barr levels (Reaction, Learning, Behavior, and—at follow-up—Results), plus cross-cutting suggestions; (4) iterative theme review with explicit checks for consistency across timepoints (from intention to implementation to outcomes); (5) definition and naming of themes reflecting participants’ language where appropriate (e.g., “mental-health slots,” “shared folder”), with exemplar cases identified (e.g., use of the M-CHAT); and (6) integrated reporting across timepoints.

The analysis aimed to preserve participants’ voices while summarizing findings to inform refinement and sustainability of future course iterations and related service-delivery adjustments.

The combined analysis of the post-course (4 August 2025) and 3-month follow-up (3 November 2025) focus groups provides insight into immediate reactions and learning, the translation of intentions into clinic behaviors, and early signals of outcomes at patient/family and system levels. Organized by the Kirkpatrick–Barr framework, we first summarize post-course insights (reaction and learning at Levels 1–2 and intentions at Level 3) and then extend them with follow-up evidence on implemented behaviors (Level 3) and perceived results (Level 4).

#### 4.2.1 Theme: course experience and satisfaction (Level 1—Reaction)

Post-course, all five participants described the training as highly relevant and practical for everyday pediatric practice, with attention-deficit/hyperactivity disorder (ADHD) and autism spectrum disorder (ASD) sessions most frequently cited as useful. The interactive, case-based approach and in-person format were consistently preferred over online delivery. Participants requested additional case-based learning and brief quizzes for knowledge reinforcement. At follow-up, clinicians reaffirmed the ongoing relevance of the course and emphasized that case-based discussions remained the most transferable element to real clinics; they expressed a preference for periodic “booster” case discussions to sustain momentum and address emerging challenges.

#### 4.2.2 Theme: learning and confidence (Level 2—Learning)

Post-course, participants reported increased confidence in identifying and initiating management for behavioral and emotional difficulties, clearer thresholds for referral to specialized services, improved readiness to report child protection concerns, and a more biopsychosocial assessment style. By follow-up, this confidence was maintained and became anchored in routine use of structured tools—specifically M-CHAT, the VARS, and the SDQ—alongside clearer communication with families and schools regarding findings and reasons for referral to specialized services.

#### 4.2.3 Theme: application—from intention to behavior (Level 3—Behavior)

Immediately after the course, pediatricians intended to implement M-CHAT, VARS, and SDQ; create a shared folder of screening tools; and incorporate more parent psychoeducation into visits. They anticipated barriers related to short consultation times, parental denial or resistance, limited community awareness, and access constraints to specialist services. At 3 months, clinicians reported that routine age-appropriate screening with M-CHAT, VARS, and SDQ had been integrated into practice and that structured electronic medical record (EMR) referrals to mental health services were being used. Parent psychoeducation at the point of care had become more consistent, with screening results improving acceptance of referrals. One exemplar case involved a child with limited socialization who screened M-CHAT = 9, prompting timely referral and parental acceptance. Participants highlighted ready access to tools, administrative encouragement for a systematic approach, and a clear EMR pathway as key enablers. To mitigate time pressure, they proposed introducing 30-min “Pediatric Mental Health” appointment types, flagging such slots at booking, and piloting pre-visit digital screening (e.g., SDQ via SMS) to conserve in-clinic time. Persistent constraints included the standard 10-min slot length, ongoing service-access limitations, and variable community/teacher awareness, which collectively moderated the pace and scope of practice change.

#### 4.2.4 Theme: suggestions for improvement and sustainability

Post-course recommendations focused on expanding content (bullying, aggression, eating disorders, and personality traits/disorders), increasing in-person sessions, integrating quizzes, and offering the course twice per year. Participants also advocated for joint pediatric–psychiatry clinics to provide hands-on collaborative experience. At follow-up, these ideas were further operationalized: clinicians requested monthly case-discussion boosters (virtual or in-person) to consolidate skills; trial joint pediatric–psychiatry clinics to model shared care and accelerate local upskilling; embed 30-min mental-health slots, pre-visit screeners, and a clinic toolkit (M-CHAT, VARS, SDQ, and brief psychoeducation handouts); and develop teacher education sessions and parent information on services and referral pathways to align expectations and reduce delays.

#### 4.2.5 Theme: early outcomes at 3 months (Level 4—Results)

While the focus group was exploratory and qualitative—based on participants’ self-reported experiences—clinicians described early, perceived benefits. They reported that families seemed more satisfied, which they attributed to clearer explanations and education, routine use of structured screening, and increased clinician confidence. At a system level, participants noted more school-initiated requests for support resulting in earlier identification of comorbidities (e.g., behavioral difficulties in children with speech delay prompting timely referral). At the same time, they did not yet feel ready to manage moderate or complex presentations independently, underscoring the need for stepped-care pathways and ongoing collaboration. Participants also highlighted an external gap: teachers require further preparation to support behavioral challenges, reflecting needs beyond the clinic’s remit. These observations should be interpreted as early signals rather than definitive outcomes.

Overall synthesis. The post-course group demonstrated strong acceptability and perceived learning; by 3 months, these gains translated into routine screening, EMR-facilitated referrals, and more consistent parent psychoeducation, with early signals of improved family experience and school engagement. Enduring constraints—particularly time, geographic access to specialist care, and school/community readiness—shape sustainability. The combined findings support targeted operational adjustments (e.g., flagged 30-min slots and pre-visit screening) and collaborative supports (booster sessions and joint clinics), alongside community and teacher education, to deepen and sustain impact.

## 5 Discussion

This study evaluated an 8-week case-based child and adolescent mental health course for pediatricians in the United Arab Emirates using a prospective mixed-methods design. By integrating pre-course, post-course, and follow-up survey findings with qualitative feedback, the study examined changes in participants’ knowledge, confidence, perceived clinical practice, and experience of the course.

Overall, the findings suggest that the course was feasible, highly acceptable, and associated with meaningful short-term and early sustained changes. At Kirkpatrick–Barr Level 1 (Reaction), participants reported very high satisfaction and strong perceived relevance to practice. At Level 2 (Learning), group-level analyses showed large improvements in confidence across multiple targeted competencies, with particularly large effects for conducting structured mental health screening and managing mild to moderate mental health problems. At Level 3 (Behavior), most clinicians reported adopting routine screening practices and using standardized tools (e.g., VARS, M-CHAT, SDQ) by follow-up. At Level 4 (Results), participants described early signals of improved care (e.g., more timely referrals, improved symptoms, and enhanced family engagement), alongside initial system-oriented changes such as the introduction of new tools/protocols and more structured collaboration.

The pattern of findings—high satisfaction, large confidence gains, and reported practice adoption at follow-up—likely reflects the “practice-facing” design of the intervention. The course deliberately combined short evidence-informed teaching with locally relevant vignettes, guided case discussion, and explicit strategies for assessment, management, and collaboration. This design aligns with continuing professional development guidance emphasizing interactive, needs-based learning approaches that are more likely to support behavior change than purely didactic formats Accreditation Council for Continuing Medical Education (ACCME), (12).

Although participants expressed high satisfaction with the course, the findings also suggest early improvements in self-reported clinical care. These included increased confidence in assessment and referral, greater attention to psychosocial factors in pediatric presentations, and examples of applying screening tools and earlier intervention in practice. Accordingly, the value of the course appears to extend beyond participant satisfaction to potentially meaningful changes in day-to-day clinical work.

Importantly, the study’s mixed-methods data converge: post-course intentions included routine screening and use of standardized tools, and follow-up reports suggested that many of these intentions translated into practice changes (e.g., 5/8 reporting standardized tool use; workflow integration and case discussions). Qualitative feedback highlighting the value of practical tools and requests for ongoing case-based support is consistent with the notion that confidence gains may be most durable when paired with reinforcement, mentorship, and system supports. These findings align with the broader capacity-building literature indicating that interactive training often produces the most consistent gains in clinician competence and healthcare outcome (22, 23). In a Canadian systematic review of child and adolescent mental health capacity-building initiatives for primary care providers, Gotovac and colleagues noted substantial variability in programs and a frequent reliance on self-report outcomes, emphasizing the need for more rigorous, multi-level evaluation (7).

This study’s outcomes are also directionally consistent with controlled evidence from the Practitioner Training in Child and Adolescent Psychiatry (PTCAP) cluster-randomized trial, where physicians showed significantly improved confidence in managing mental health conditions among children and adolescents after an 8-h CME course (13). Compared with shorter interventions, this 8-week case-based format adds an element of spaced learning and repeated case application that may support early behavior change, reflected here in follow-up uptake of structured tools and screening routines.

Beyond PTCAP, longer-term evaluations of training efforts have also shown that clinician training can translate into sustained practice change. For example, a training program focused on screening, assessment, and treatment of adolescent depression reported increased provider screening rates over extended follow-up (24). Similarly, case-based models [e.g., Project ECHO (Extension for Community Healthcare Outcomes) approaches] have reported improvements in clinician knowledge and self-efficacy, alongside signals of practice change and service impact, such as increased treatment engagement and reductions in emergency department visits in some implementations (25–27). Taken together, this study’s data fit well within the international pattern: interactive, case-based training is consistently associated with improved clinician confidence, and when paired with practical tools and collaborative pathways, may support early adoption of more structured frontline care.

From a health-system perspective, the findings are timely for the UAE, where recent work has documented training gaps among primary care clinicians and recommended practice-oriented training and integrated care pathways to strengthen early detection and management (4). The follow-up data—particularly adoption of standardized tools (VARS/M-CHAT/SDQ) and reported refinement of referral pathways —suggest that relatively low-resource educational interventions can catalyze concrete workflow changes when content is locally relevant and aligned with specialist collaboration. Integration of validated screening tools into electronic medical record (EMR) systems may further enhance sustainability by supporting routine use, standardized documentation, and timely referrals within primary care workflows.

For scale-up, several implementation supports follow logically from the participants’ reported barriers and recommendations: protected time within clinics, easier access to child psychiatry consultation, and periodic refresher or case-review sessions. Although conducted at a single site, the curriculum was designed around common pediatric mental health presentations, collaborative-care principles, and established educational frameworks, suggesting potential adaptability to similar service settings. Future dissemination across other hospitals or primary care settings would allow broader evaluation of feasibility, acceptability, and generalizability. A practical next iteration could combine the current course with a light-touch longitudinal component (e.g., monthly case conferences), explicitly targeting maintenance of skills and bridging training to measurable service outcomes.

This study has several strengths, including prospective measurement across three time points, explicit use of a recognized evaluation framework, and mixed-methods triangulation that helps interpret not only “if” change occurred but “how” it may have occurred. In addition, the intervention was implemented in a real-world service setting with high participation across time points, enhancing feasibility and practical relevance. The inclusion of follow-up qualitative exploration also strengthened interpretation of sustainability by capturing barriers and facilitators to implementation beyond immediate post-course perceptions.

Findings should be interpreted in light of several limitations. First, the small single-site cohort limits generalizability, and the findings should be interpreted as preliminary. Second, most outcomes relied on self-reported measures of knowledge, confidence, practice change, and perceived patient or system impact, which are vulnerable to recall and social desirability bias and do not necessarily reflect objective clinical behavior or patient outcomes. In particular, Levels 3 and 4 of the Kirkpatrick–Barr framework were assessed through clinician report rather than objective indicators such as electronic health record data, observed practice, referral appropriateness, or standardized patient outcome measures. These findings should therefore be interpreted as preliminary and perceived signals of impact rather than definitive evidence of higher-level outcomes. In addition, only a subset of participants could be reliably matched across time for paired analyses due to missing identifying codes on follow up, reducing statistical power for within-person change tests; for example, the paired screening uptake signal was strong but underpowered for conventional significance thresholds.

Future work would benefit from larger multi-site cohorts, improved linkage of repeated measures, longer follow-up, and objective outcomes (e.g., electronic health record indicators, referral appropriateness, wait-time metrics, patient-reported outcomes). A controlled or stepped-wedge design would further strengthen inference about effectiveness while remaining feasible in service settings.

## 6 Conclusion

An 8-week case-based child and adolescent mental health course for pediatricians in the UAE was highly acceptable and associated with substantial improvements in self-reported confidence, alongside early evidence of practice adoption at 3–6-month follow-up (including increased use of standardized screening tools and structured workflows). While findings are preliminary due to the pilot single-cohort design, small sample, and reliance on self-report, they support the feasibility and potential value of structured, locally tailored capacity-building interventions to strengthen frontline pediatric mental health care within collaborative-care pathways. Larger, multi-site evaluations incorporating objective clinical and system outcomes are warranted.

## Data Availability

The original contributions presented in this study are included in the article/supplementary material, further inquiries can be directed to the corresponding authors.
